# The Pleiotropic Effects of Carbohydrate-Mediated Growth Rate Modifications in *Bifidobacterium longum* NCC 2705

**DOI:** 10.3390/microorganisms11030588

**Published:** 2023-02-26

**Authors:** Stéphane Duboux, Solenn Pruvost, Christopher Joyce, Biljana Bogicevic, Jeroen André Muller, Annick Mercenier, Michiel Kleerebezem

**Affiliations:** 1 Société des Produits Nestlé SA, Nestlé Research, Route du Jorat 57, CH 1000 Lausanne, Switzerland; 2 Host-Microbe Interactomics Group, Wageningen University & Research, De Elst 1, 6708 WD Wageningen, The Netherlands

**Keywords:** bifidobacteria, carbon metabolism, growth rate, catabolite repression, stress resistance

## Abstract

Bifidobacteria are saccharolytic bacteria that are able to metabolize a relatively large range of carbohydrates through their unique central carbon metabolism known as the “bifid-shunt”. Carbohydrates have been shown to modulate the growth rate of bifidobacteria, but unlike for other genera (e.g., *E. coli* or *L. lactis*), the impact it may have on the overall physiology of the bacteria has not been studied in detail to date. Using glucose and galactose as model substrates in *Bifidobacterium longum* NCC 2705, we established that the strain displayed fast and slow growth rates on those carbohydrates, respectively. We show that these differential growth conditions are accompanied by global transcriptional changes and adjustments of central carbon fluxes. In addition, when grown on galactose, NCC 2705 cells were significantly smaller, exhibited an expanded capacity to import and metabolized different sugars and displayed an increased acid-stress resistance, a phenotypic signature associated with generalized fitness. We predict that part of the observed adaptation is regulated by the previously described bifidobacterial global transcriptional regulator AraQ, which we propose to reflect a catabolite-repression-like response in *B. longum*. With this manuscript, we demonstrate that not only growth rate but also various physiological characteristics of *B. longum* NCC 2705 are responsive to the carbon source used for growth, which is relevant in the context of its lifestyle in the human infant gut where galactose-containing oligosaccharides are prominent.

## 1. Introduction

Bifidobacteria are important members of the human gut microbiota that particularly strive in early infancy [1]. It has been proposed that their presence in that stage of life plays a critical role in the maturation of the newborn’s immune system as several studies have linked low abundance of members of this genus in the infant gut to later in life immune disorders such as atopy [2] or asthma [3]. Early-life *Bifidobacterium* dominance has also been advocated to be linked with reduced risk of acquisition of antimicrobial resistances [4] or obesity [5] later in life.

Reflecting their adaptation to the human gut environment, bifidobacteria harbor an extensive carbohydrate-active enzyme repertoire that supports their capacity to effectively breakdown and metabolize a large range of carbohydrate substrates [6,7]. In particular, the predominance of *Bifidobacterium* in early infancy has been attributed to their capacity to grow on Human Milk Oligosaccharides (HMO) that besides lactose dominate the available sugars found in human milk [8]. Strains belonging to *B. longum* subsp. *infantis* have been shown to be particularly adapted to import and hydrolyze a range of fucosylated or sialylated HMOs [9,10,11], and the capacity to metabolize the neutral Lacto-N-Tetraose (LNT) is considered to be broadly conserved in all subspecies of *B. longum* [7,12]. In members of the *Bifidobacterium* genus, carbohydrates are processed through a relatively unique central carbon metabolic pathway, the so-called “bifid-shunt”, which involves the bifunctional xylulose 5-phosphate/fructose-6-phosphate phosphoketolase enzyme (F6PPK) [13]. As an example, glucose, fructose, mannose, xylose, ribose and galactose were all shown to be catabolized via the “bifid-shunt” in *B. longum* NCC 2705 [14].

Carbohydrate metabolism is the predominant source of ATP for bifidobacteria [15], and carbon import and metabolic fluxes should be tightly controlled to allow nutrient adaptation and maintenance of fitness under fluctuating environmental conditions. In gram-positive bacteria, catabolite repression (often mediated by glucose or lactose) has been shown to control a wide array of processes, including the repression of expression of import functions for alternative sugar sources and regulation of central carbon metabolism flux [16]. The same regulation process has not been extensively investigated in bifidobacterial strains, although several manifestations of glucose-mediated catabolite repression have been observed, suggesting its existence in this genus as well. For example, glucose was demonstrated in *B. longum* to repress the production of *Frk*, a fructokinase required for the catabolism of fructose [17]. Recently, we demonstrated that glucose and galactose are sequentially metabolized in *B. longum* NCC 2705 and that the presence of residual glucose inhibited the consumption of galactose in that strain [18]. In *B. lactis*, glucose was shown to repress the expression of sucrose utilization genes [19]. We recently demonstrated that in *B. longum* NCC 2705 growth on glucose inhibited production of the serine protease inhibitor (serpin) which was relieved when the strain was grown on different sugars [20]. In most gram-positive bacteria, catabolite repression is controlled by a protein complex of histidine phosphocarrier protein (HPr) and catabolite control protein A (CcpA), a LacI-type regulator that can bind to specific DNA motifs (so-called *cre*-elements) and inhibit or activate transcription of downstream genes [21,22]. Of note, recognizable orthologues of the CcpA protein are absent in bifidobacteria and typical genome-wide catabolite repression mechanisms have not been described in detail for the members of this genus. Nevertheless, two LacI-type transcriptional regulators, AraQ and MalR1, were demonstrated to control a large set of genes spread over the genome of *B. breve* UCC 2003. Both proteins were predicted to regulate a range of transcriptional factors in this strain, to repress transcription of a number of genes related to sugar import, and to activate transcription of genes related to central carbon metabolism including genes involved in the “bifid-shunt” [23,24]. Therefore, AraQ and MalR1 could represent the central regulators of the bifidobacterial variant of the canonical CcpA-mediated catabolite regulation mechanism.

In most prokaryotes, consumption of glucose is coupled to fast growth, which at least in part results from the tight catabolite repression control [16]. Relief of catabolite repression enables bacteria to consume a larger variety of carbon sources often resulting in a reduction of growth rate [25]. These slow-growing bacterial cells are not only better prepared for the utilization of alternative carbon substrates, but are also displaying increased resistance to stress [26,27,28]. These effects were predominantly demonstrated using cells grown at different rates in carbon-limited chemostats, but recent evidences suggest that the same outcome can be obtained by varying the carbon source used for growth [29]. It has previously been shown that bifidobacteria display different growth rates depending on the available carbohydrate substrate. For example, most tested bifidobacterial strains displayed a reduced growth rate on galacto-oligosaccharides (GOS) as compared to glucose [29], whereas the monosaccharide galactose induced a substantial growth rate reduction in *B. longum* and *B. adolescentis* strains [30]. However, the overall physiological impact of sugar-dependent growth rate-modifications and the possible involvement of catabolite-repression-like control mechanisms remain unresolved in bifidobacteria.

The present study aimed to investigate whether carbohydrate induced growth rate variation may influence the physiology of *B. longum* NCC 2705. Glucose and galactose were selected not only for their capacity to modulate the growth rate of the strain, but also because they are the common carbon sources in bifidobacterial growth media [31] as well as important substrates for bifidobacteria in the infant colon as they are core-constituents of HMOs [8]. We show that growth on glucose and galactose led to distinct metabolic and physiological changes and that this was associated with substantial genome-wide transcriptional modifications. Overall, this study provides a further demonstration of the pleiotropic impact of specific carbohydrate substrates on the physiology of this saccharolytic bacterium, which support the existence of a catabolite-repression-like mechanism in *B. longum* NCC 2705.

## 2. Materials and Methods

### 2.1. B. longum NCC 2705 Cultivation

*B. longum* NCC 2705 was obtained from the Nestlé Culture Collection (NCC) (Nestlé Research, Lausanne, Switzerland). The strain was grown in a modified de Man, de Rogosa & Sharpe medium lacking carbohydrates (MRS-C) (10 g/L bacto proteose peptone, 3, 5 g/L bacto yeast extract, 1 g/L Tween 80 (Chemie Brunschwig, Basel, Switzerland); 2 g/L di-ammonium hydrogen citrate, 5 g/L sodium acetate, 0.1 g/L magnesium sulphate, 0.05 g/L manganese sulfate, 2 g/L di-sodium phosphate and 0.5 g/L cysteine (Sigma-Aldrich Chemie GmbH, Buchs, Switzerland). MRS-C was supplemented with 1% galactose or 1% glucose (Sigma-Aldrich Chemie GmbH) using filter-sterilized (0.22 μm) stock solutions prepared at 100 g/L in water. Overnight cultures were inoculated at 2% in fresh medium and growth was performed at 37 °C in 2 L DasGip bioreactors (Eppendorf AG, Hamburg, Germany), using 500 mL working volume. Anaerobiosis was achieved by CO_2_ flux in the headspace, and agitation was maintained at 600 rpm. pH was monitored throughout the fermentation and samples were taken at regular interval to measure Optical Density (OD measured at 600 nm) and enumerate Colony Forming Units (CFU) using standard MRS-agar supplemented with 0.05% cysteine (48 h anaerobic incubation at 37 °C). In addition, at the same timepoints cells were collected by centrifugation (3000× *g*, 10 min at 4 °C) and resuspended in sterile PBS supplemented with 15% glycerol (Sigma-Aldrich Chemie GmbH). Aliquots were stored at −80 °C until further analysis. Every fermentation condition was run in biological triplicate.

### 2.2. Growth Data Analysis

Raw data obtained from the above fermentations were analyzed as follows. Maximal acidification slope was obtained by modeling inversed pH values for each biological replicate with a logistic growth model in GraphPad Prism (v8.1.1, GraphPad Software Inc., San Diego, CA, USA). The same modelling strategy was applied to the OD 600 nm values to obtain the maximal growth rate in each fermentation condition. CFU/mL and OD values obtained in each biological replicates before the decrease in viability (in each bioreactor) were used to calculate the average CFU:OD ratios. For both glucose and galactose conditions, average OD values were multiplied by the corresponding average CFU:OD ratio to obtain theoretical CFU/mL. A logistic growth model was then applied on the theoretical CFU/mL values, to finally estimate the average cell doubling time for each condition.

### 2.3. Microscopic Observations

Samples collected at the different stages of growth (mid-exponential, early and late stationary phases) were fixed using a 10% solution of Nigrosin (Sigma-Aldrich Chemie GmbH) and analysed by confocal microscopy (Olympus Life Science, Hamburg, Germany). For each growth phase and growth condition, the images were analyzed using the cellSens Entry software (v1.16) (Olympus Life Science) to determine the cell size of a minimum of 50 individual cells. All data points were then merged (per growth condition) to calculate an averaged cell length and the respective standard error of the mean (SEM) in GraphPad Prism (v8.1.1, GraphPad Software Inc.).

### 2.4. RNA Isolation and Sequencing

Cells were harvested from 2 mL culture samples collected during mid-exponential growth (OD_600_ ~0.6–0.8) by centrifugation (3000× *g*, 3 min at 4 °C). RNA was isolated from each biological triplicate using the miRNeasy kit (QIAGEN AG, Hombrechtikon, Switzerland). RNA concentration was measured using the Quant-it Ribogreen RNA kit (Thermo Fisher Scientific, Waltham, MA, USA) and RNA quality was evaluated using the Agilent 2100 Bioanalyser system with the Agilent RNA 6000 nano kit (Agilent Technologies, Santa Clara, CA, USA). Library preparations were performed with the Prokaryotic AnyDeplete kit, which provides all reagents necessary to produce ready to sequence RNA libraries (Protocol M01502 v1, NuGEN Technologies Inc., Redwood City, CA, USA). The Prokarotic AnyDeplete workflow is a follows: 250 ng of total RNA was used as input to produce cDNA, followed by an enzymatic fragmentation to a target size of around 200 pb. End-repair of the cDNA was performed, and specific adaptors provided with the kit (8 bases index allowing sample multiplexing for Illumina sequencing) were added by ligation. A strand selection step (to retain RNA strand information) was followed by a ribodepletion step aiming at eliminating unwanted rRNA transcript sequences. Finally, 18 PCR cycles were used for the final amplification of the libraries using specific primers provided in the AnyDeplete kit. Obtained DNA fragments were then purified with a 1:1 ratio of AMPURE XP beads (Beckman Coulter Life Sciences, Indianapolis, IN, USA). Libraries were checked on a Labchip GX touch with DNA High Sensitivity Reagent Kit (PerlinElmer Inc., Waltham, MA, USA). All samples were finally sequenced on an Illumina HiSeq 2500 with Rapid V2 chemistry (Illumina Inc., San Siego, CA, USA), applying 200 cycles (paired-end reads PE100). Flowcell was loaded with 10 pM of DNA and with 2% PhiX control v3 library (internal control enabling quality check of the sequencing run; Illumina Inc.). All raw sequencing data were deposited at NCBI (Sequence Read Archive) in the Bioproject PRJNA936080.

### 2.5. Transcriptome Analysis

A dedicated RNAseq analysis pipeline for prokaryotes [32] was used for the analysis of the obtained *B. longum* NCC 2705 transcriptional data. Reference genome was obtained from NCBI RefSeq (NC_004307.2). Following mapping with HISAT2 (version 2.1.0) [33], reads were softclipped and mapping quality set to 0 for unmapped reads (picard-tools version 1.119, CleanSam; https://gatk.broadinstitute.org/hc/en-us/articles/360036885571-CleanSam-Picard-; accessed on 19 August 2019). The resulting bam files were then merged using samtools [34] (version 1.4) prior to read counting using FADU [32] (version 1.7). Counts obtained were first filtered to keep only genes having at least 5 read-counts in at least 2 samples. DEseq2 R package [35] was used for both normalization (performed using RLE; Relative Log Expression) and differential expression analysis. Differentially expressed genes (DEG) were filtered based on their False Discovery Rate corrected p-values (FDR < 0.05) and used for Gene Ontology (GO) enrichment, which was performed with the BINGO Cytoscape plugin [36], using the *B. longum* NCC 2705 reference proteome available at Uniprot as basis for annotation [37]. A central carbon metabolism pathway map was reconstructed in Cytoscape based on different KEGG maps (00052, galactose metabolism; 00030, pentose phosphate; 00010, glycolysis; 00620, pyruvate metabolism). Similarly, a map of the different sugar transporter systems present in the strain was reconstituted starting from Parche et al. [38]. Finally, the log2FC values obtained previously were mapped on the list of AraQ and MalR1 regulons retrieved from the RegPrecise database [39], and used to color the constructed maps in Cytoscape.

### 2.6. Acetate, Lactate, Ethanol and Formate Quantifications

The (relative) levels of formate, acetate, lactate and ethanol in spent culture supernatants were determined by NMR spectroscopy. To this end, *B. longum* NCC 2705 was cultured to mid-exponential phase on glucose and galactose in tightly closed tubes in triplicate. Cells were removed by centrifugation (3300× *g*, 2 min at 4 °C) and the culture supernatant was filtered through a 0.22 μm polyether sulfone filter and stored at −20 °C until further analysis. For NMR spectroscopy samples were diluted 1:1 in a 10% D_2_O/90% H_2_O solution. Then, 1D Nuclear Overhauser Effect Spectroscopy (NOESY) spectra were measured on a 600 MHz NMR spectroscopy (Bruker BioSpin, Billerica, MA, USA) equipped with a 5 mm cryo-probe at a temperature of 300 K. The ^1^H-^1^H mixing time was 10 ms. A saturation of 50 Hz was applied on water during relaxation delay and mixing. Quantum Mechanics-based 1H full Spin Analysis with the Cosmic Truth software (NMR Solutions Ltd., Kuopio, Finland) was employed for data analysis and peak assignments (for details see [40,41]). Peak intensities of the metabolic end-products were quantified in comparison to spiked-in standard of ^13^C labelled acetate. Average and standard deviations were calculated in GraphPad Prism (v8.1.1, GraphPad Software Inc.).

### 2.7. Flow Cytometry Analysis

Flow cytometry analyses were performed with a Beckman Coulter Cytoflex S (Beckman Coulter, Brea, CA, USA) equipped with four lasers. Bacterial cell staining was performed using 1.5 μM propidium iodide (PI) and 5 μM 5-carboxyfluorescein diacetate acetoxymethyl ester (CFDA) with an incubation of 10 min at room temperature before analysis. Forward scatter acquisition threshold was set at 1000 in order to capture all cells, including the small galactose-grown ones. Cell size was estimated using the Forward Scatter signal (FSC). Cell permeability was evaluated through PI (Thermo Fisher Scientific AG) staining and measured using the ECD channel (excitation at 561 nm, emission at 610/20 nm). Metabolic activity was evaluated using the cell-permeant esterase substrate CFDA (Thermo Fisher Scientific AG) measured using the FITC detector (excitation at 488 nm, emission at 525/40 nm). Acquisitions were processed with the Beckman-Coulter CytExpert Acquisition software version 2.3, followed by further analysis with the FCSExpress software version 7.01 (De Novo Software, Pasadena, CA, USA). Statistical analysis of population distribution was performed in GraphPad Prism (v8.1.1, GraphPad Software Inc.), using a Welch-*t* test.

### 2.8. Translationally Blocked Acidification Assay

The assay used was adapted from Nugroho et al. [42], and aimed to determine the acidification capacity of translationally blocked glucose or galactose-grown *B. longum* NCC 2705 using a set of different carbohydrate substrates. The assay was performed in microwell plates under anaerobic conditions, in biological triplicate. Protein translation was blocked using erythromycin and once the bacteria switched to the new carbohydrates, acidification was followed over the incubation period using carboxyfluorescein. The assay medium was adapted from MRS-C by removing yeast extract, to avoid contamination steming from the residual sugars contained in this ingredient. For this assay, *B. longum* NCC 2705 was grown to the mid-exponential phase on MRS-C supplemented with either glucose or galactose as a sole carbon source. Five ml of cultures were harvested by centrifugation (2 min at 3300× *g*), washed with 1 volume of sterile PBS and resuspended at an OD_600_ of 0.6 (final OD in the well 0.3) in 2× concentrated assay medium without sugar (for 1×: 5 g/L bacto proteose peptone, 1 g/L Tween 80 (Chemie Brunschwig); 2 g/L di-ammonium hydrogen citrate, 5 g/L sodium acetate, 0.1 g/L magnesium sulfate, 0.05 g/L manganese sulfate, 2 g/L di-sodium phosphate, 0.5 g/L cysteine, 5 μg/mL erythromycin, 10 μM carboxyfluorescein (Sigma-Aldrich Chemie GmbH)). Notably, the Minimum Inhibitory Concentration (MIC) of erythromycin for NCC 2705 is of 0.03 μg/mL (166 times less than the concentration used in the assay). Then, 100 μL of cell suspension was added to the different wells, which were all prefilled with 100 μL of the different 2× filtered sterile carbohydrate solutions (final sugar concentration in the well of 5 g/L; glucose, galactose, fructose, raffinose, maltose, lactose, sucrose, arabinose (Sigma-Aldrich Chemie GmbH), NutraFlora scFOS (Ingredion Korea Inc., Gyunggi-do, Korea), Vivinal GOS (FrieslandCampina DOMO Amersfoort, The Netherlands), Lacto-N-tetraose LNT (Glycom A/S, Lyngby, Denmark)). All solutions (2× concentrated assay medium, 2× carbohydrate solutions) were prewarmed at 37 °C in a water bath before starting the assay. Finally, the 96-microwell plate containing the translationally blocked cells mixed with the different sugars (200 μL in each well) was sealed in a plastic bag containing an AGELESS ZPT mini anaerobic sachet (Mitsubishi Gas Chemical Gmbh, Dusseldorf, Germany) and immediately transferred in a Varioskan spectrophotometer (Thermo Fisher Scientific AG), maintained at a temperature of 37 °C. Fluorescence was measured every 10 min (excitation at 485 nm, emission at 535 nm) over a limited period of 200 min to avoid measuring an acidification coming from a possible translational readaptation of the strain. A logarithmic curve was then fitted to inversed individual acidification curves in Excel for each individual biological replicate. Acidification rates were extracted by calculating the slope of each individual curve and triplicate values were then transferred to GraphPad Prism (v8.1.1, GraphPad Software Inc.) where they were subjected to an unpaired multiple *t*-test comparison.

### 2.9. Loss of Metabolic Activity upon Exposure to Low pH

Frozen mid-exponential or stationary phase harvested cells of *B. longum* NCC 2705, stored at −80 °C in a PBS suspension containing 15% glycerol were used as starting material. Initial trials aimed to select an optimal pH under which a significant fraction of the cells in the suspension lost their metabolic activity (as reflected by the loss of esterase activity measured using CFDA) during a stress exposure period of 18 min. To assess the appropriate incubation pH to achieve the desired assay conditions, glucose grown stationary phase harvested cells were thawed on ice, washed in one volume of sterile PBS (centrifugation 2 min, 3300× *g*), and resuspended to an OD of 1.0 in PBS adjusted at different pH values (pH 2.9, 3.0, 3.2, 3.5) using 1 M hydrogen chloride (HCl). The obtained solution was distributed in 100 μL aliquots and incubated at 37 °C for a period of 18 min. An aliquot was collected every 3 min, and 900 μL of PBS (pH 6.7) containing 1.5 μM PI and 5 μM CFDA were added to stop the pH induced loss of metabolic activity. After an incubation of 10 min at room temperature permeability and metabolic activity of at least 5000 individual cells was measured by flow cytometry (see above) at each timepoint and for each condition. The increase in non-metabolically active cell population fractions (CFDA-) over time was then modelled in GraphPad Prism (v8.1.1, GraphPad Software Inc.) using a logistic growth curve, as recommended by de Besten et al. [43]. Loss of metabolic activity in glucose and galactose-grown cultures (mid-exponential and stationary) upon exposure to a pH of 2.9 was then pursued using a similar protocol, in biological triplicate. The triplicate values obtained for the glucose and galactose conditions were compared to each other at each time point using a multiple unpaired *t*-test in in GraphPad Prism (v8.1.1, GraphPad Software Inc.). Additionally, frozen cells were compared to freshly obtained cultures to ensure that the freezing process did not impact the resistance phenotypes.

## 3. Results

### 3.1. The Distinct Growth Attributes of B. longum NCC 2705 Cultured on Glucose or Galactose as Sole Carbon Source

**Figure 1:**
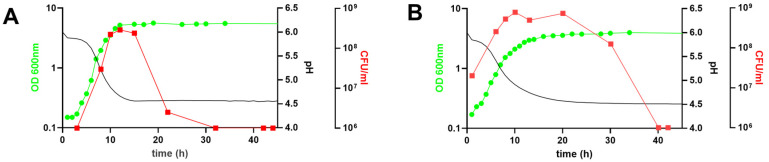
Growth characteristics of *B. longum* NCC 2705 grown in DasGip bioreactors with 1% of glucose (**A**) or 1% of galactose (**B**). Optical density measured at 600 nm (OD; green circles), cell concentration measured by Colony Forming Unit (CFUs, red squares) and pH (black line) are depicted for each condition.

**Figure 2:**
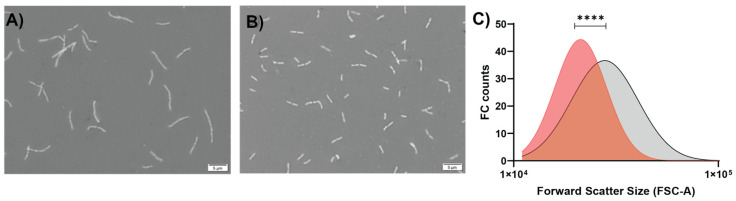
Confocal microscopic pictures of cells of *B. longum* NCC 2705 grown in DasGip bioreactors with 1% of glucose (**A**) or 1% of galactose (**B**). Panel (**C**) shows the cell size of both cultures (galactose in red, glucose in grey) represented by the forward scatter signal obtained by flow cytometry. Statistical analyses were performed using Welch-*t* test (**** *p* < 0.0001) on modeled population distributions.

*B. longum* NCC 2705 was grown in bioreactors, using MRS-based medium supplemented with glucose or galactose as sole carbon source. The maximal growth rate (μ) on glucose supplemented medium was 0.67 h^−1^, whereas maximal growth on galactose medium was approximately twofold slower (μ = 0.35 h^−1^). The estimated doubling time was of 1.05 h when the strain was grown on glucose, whereas it was twice slower (2.06 h) in the galactose condition. The observed growth rate was reflected by the difference in acidification rates, where glucose and galactose growing cells displayed maximal acidification rates of −0.57 and −0.39 h^−1^, respectively (Table 1). The maximum number of culturable cells (CFU/mL) in galactose-grown cultures was substantially higher (7.8E8 CFU/mL) than that obtained in glucose grown culture (2.8E8 CFU/mL). Additionally, a drastic decline in culturability (CFU/mL) was observed between 12 and 20 h of cultivation on glucose supplemented medium (Figure 1A), which coincides with the entry into the stationary phase of growth. In contrast, a similar decrease occurred between 30 and 40 h for the galactose-grown culture (Figure 1B), which may reflect the extended transition period from the exponential to the stationary phase observed for the galactose cultivated cells. Strikingly, and despite the similar final pH reached (4.5 in both conditions), the final optical density obtained in the glucose grown culture (OD_600_ = 5.6) was higher as compared to the galactose-grown one (OD_600_ = 3.9). Taken together, these data demonstrated that there was approximatively a four-times higher number of culturable cells per OD in the galactose-grown culture as compared to the glucose grown one, leading to CFU:OD ratios of 2.54 × 10^8^ and 5.93 × 10^7^, respectively (Table 1), suggesting a difference in cell size between the two growth conditions. Therefore, we evaluated the respective cell size of glucose and galactose-grown *B. longum* NCC 2705 using confocal microscopy and forward scatter values obtained by flow cytometry. These analyses showed that galactose-grown cells had a shortened rod-shape (1.57 ± 0.03 μm) as compared to the glucose grown cells (2.47 ± 0.06 μm; Figure 2A,B), irrespective of the growth phase at which these cells were harvested (Table 1). These cell-length estimations are in agreement with the significantly lower (*p* < 0.0001) forward scatter values obtained by flow cytometry analysis of the galactose-grown cells relative to their glucose grown counterparts (Figure 2C), confirming that galactose-grown cells are significantly smaller compared to glucose grown cells. These results highlight pleiotropic modifications of *B. longum* NCC 2705 characteristics induced by growth on glucose or galactose as sole carbon source.

### 3.2. Glucose or Galactose Dependent Central Carbon Metabolism Fluxes Modifications

**Figure 3:**
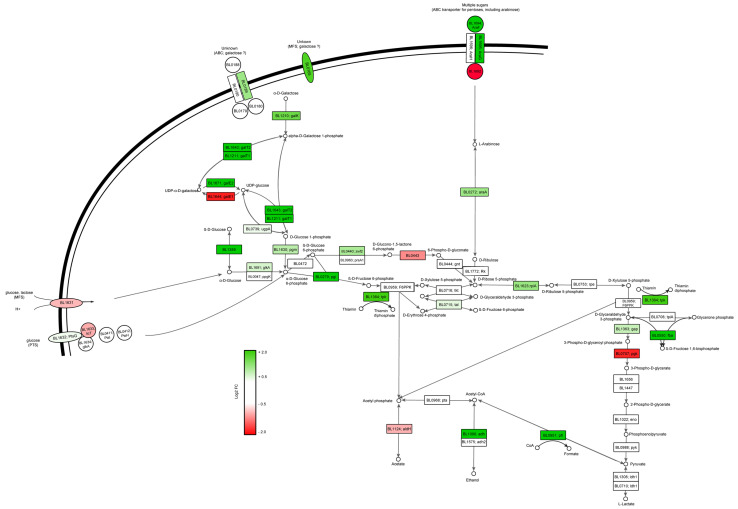
Differential expression of genes related to carbohydrate import and central carbon metabolism of *B. longum* NCC 2705 grown on galactose or glucose. Color scale represent log2 fold change in galactose:glucose (green) or glucose:galactose (red).

An initial gene ontology (GO) enrichment analysis revealed a particular enrichment in genes related to the carbohydrate metabolism (Figure S1; Tables S1 and S2), which triggered us to analyze the transcription of the genes related to the carbon import and metabolism (bifid-shunt). It revealed that growth on glucose induced the expression of glucose import systems (*Bl1631* (MFS) and *BL1633* (part of PTS)), enzymes involved in glucose-6-phosphate conversion (*BL1631*; *6PGL*), pyruvate and acetate formation (*BL0707* (*pgk*) and *BL1124* (*aldH*)), the *galE1* gene (*BL1644*) encoding the conversion of UDP-glucose to UDP-galactose, as well as the ATP binding function (*BL1692*) of the ABC transport system that was previously annotated to import arabinose [44] (Figure 3). In contrast, growth on galactose induced the expression of a substantially larger number of functions, including the relatively high-level induction of carbohydrate transporters (ABC (*BL0189*) and an MFS transport system (*BL0165*), upregulated 3.0 and 3.5 fold, respectively) that we propose to be involved in galactose import. Furthermore, functions related to the catabolism of the imported galactose were upregulated, including the conversion of D-galactose to D-glucose-6-phosphate and D-fructose-6-phosphate (*BL0739* (*ugpA*), *BL1210* (*galK*), *BL1671* (*galE2*), *BL1211* (*galT1*), *BL1643* (*galT2*), *BL1630* (*pgm*) and *BL0279* (*pgi*)). Notably, two genes (*BL1694*, *BL1695*) that encode an ABC system transporter previously suggested to be involved in arabinose transport [44] were also induced, similarly to *araA* (*BL0272*) which encodes the conversion of D-arabinose into D-ribulose, suggesting the activation of other sugar catabolism functions unrelated to galactose. Although the gene encoding F6PPK (fructose-6-phosphate-phosphoketolase, *BL0959*) that plays a central role in the bifid-shunt was not differentially expressed, the *tpk* gene (*BL1364*), responsible for the production of thiamin that acts as an essential cofactor for F6PPK, was upregulated. The activity of the F6PPK enzyme upon growth on galactose could thus be enhanced, which would be predicted to increase the levels of acetyl-phosphate in the cell. This may be relevant based on the observation that galactose growth leads to high induction of functions that utilize acetyl-phosphate as a substrate and convert it to ethanol (*BL1090*, *adh*; 4.8-fold induced) and formate (*BL0951*, *pfl*; 9.5-fold induced) (Figure 3 and Table S3).

**Figure 4:**
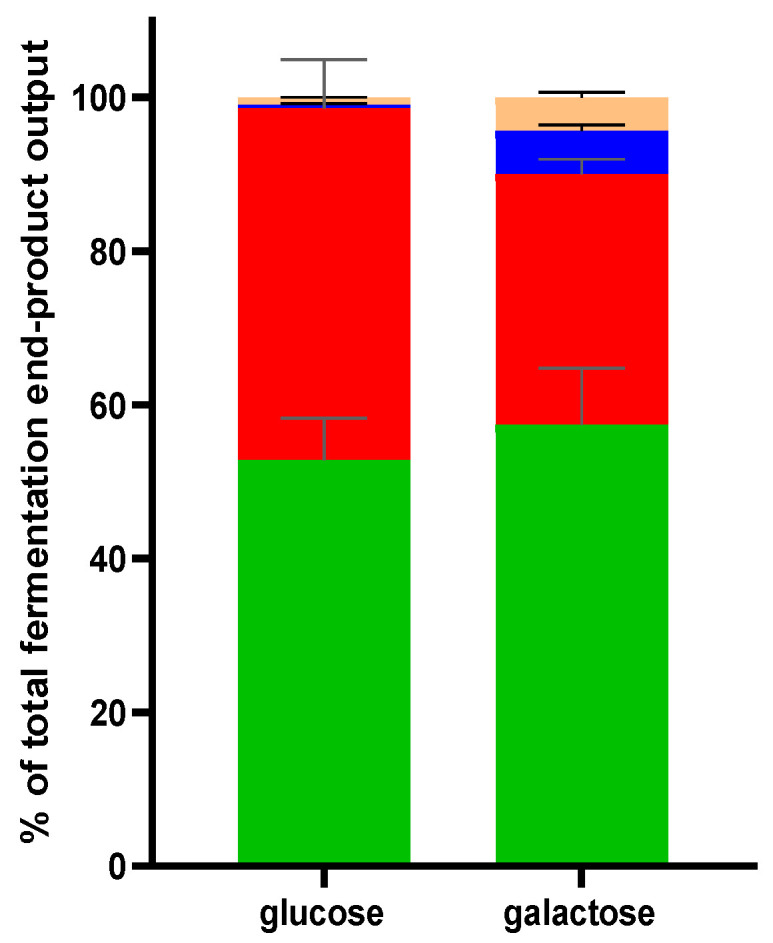
Relative levels of fermentation end products produced by *B. longum* NCC 2705 grown on glucose or galactose. Acetate (green), lactate (red), ethanol (orange) and formate (blue) levels measured in stationary phase culture supernatants.

These transcriptional changes suggest a shift in the central carbon metabolic fluxes dependent on the sugar used for growth. To evaluate this, we quantified fermentation end products of both cultures by Nuclear Magnetic Resonance (NMR) and established that formate and ethanol production increased during growth on galactose (from 0.5 to 6.1% and 0.9 to 4.8%, of the overall end-products formed, respectively), which coincided with decreased lactate production (from 45.8% to 32.6%), whereas acetate production appeared to modestly increase (52.8 to 57.3%) (Figure 4). These findings confirmed that induction of the pyruvate formate lyase (*pfl*) and alcohol dehydrogenase (*adh*) translates to increased levels of their end products (formate and ethanol, respectively). The prominent enhancement of the flux towards formate and ethanol (and to a lesser extent acetate) occurs at the expense of the flux towards lactate, involving the *ldh* encoded lactate dehydrogenase that was not differentially expressed. As a consequence, the acetate:lactate ratio shifted from 1.15 in glucose grown cells to 1.76 in galactose-grown cells of *B. longum* NCC 2705.

### 3.3. Expanded Carbohydrate Utilization Capacity in Galactose-Grown B. longum NCC 2705

In *L. lactis* and *E. coli*, growth rate reduction was shown to coincide with the production of non-essential proteins implicated in the import and metabolization of alternative sugar sources [45]. *B. longum* NCC 2705 grows slower on galactose (Table 1) and upon growth on this carbohydrate, genes implicated in transport and metabolization of another sugar (arabinose) were induced. Therefore, we evaluated in more detail to what extent genes involved in the transport of carbohydrates (beyond glucose, galactose and arabinose) were differentially expressed in the two conditions studied.

**Figure 5:**
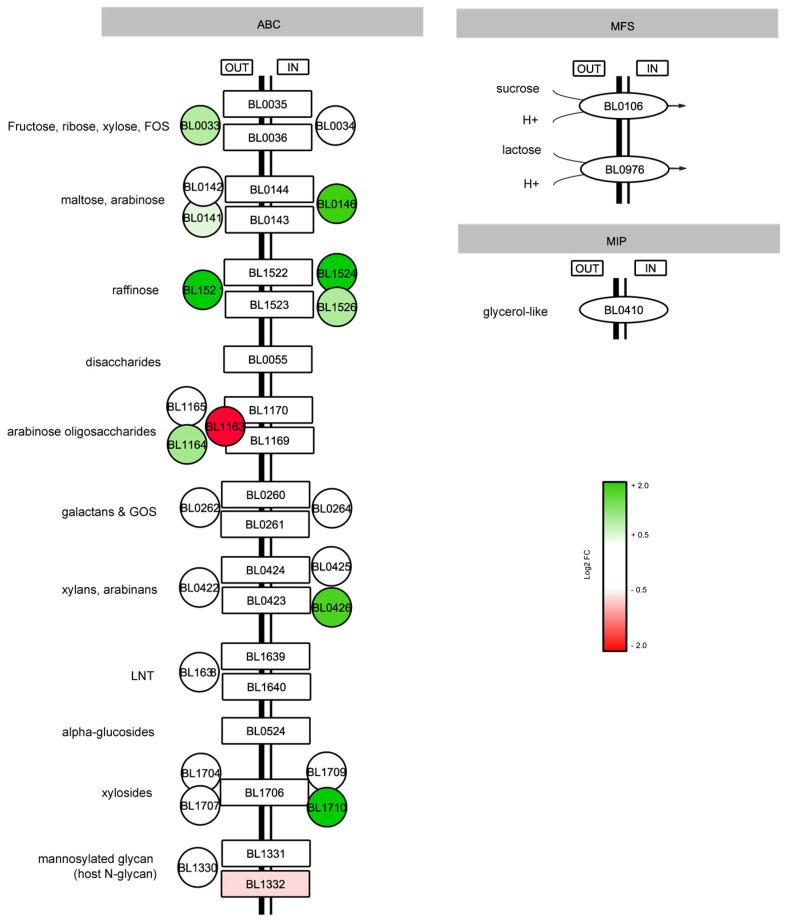
Differential expression of genes related to carbohydrate transport in *B. longum* NCC 2705 grown on galactose or glucose. Fold expression change in galactose growth induced (green) and glucose growth induced genes (red) are displayed in log2 scale.

During glucose growth, a gene involved in the transport of mannosylated oligosaccharides (*BL1332*) was slightly but significatively upregulated. In contrast, in galactose growing cells several genes predicted to be involved in the import of various carbohydrates were induced, including the transporters for fructose and FOS (*BL0033*), maltose and arabinose (*BL00141*, *BL0146*), raffinose (*BL1521*, *BL1524*, *BL1526*), xylans and arabinans (*BL0426*), and xylosides (*BL1710*) (Figure 5). Interestingly, differential but opposite regulation was observed for the two substrate-binding proteins associated with an ABC-transport system predicted to import arabinose oligosaccharides (*BL1163* induced 9.4-fold in glucose, *BL1164* induced 2.2-fold in galactose). These data suggest that relative to glucose-grown cells, the slower growing galactose cells are potentially able to utilize a larger set of carbohydrate-substrates, a phenotype adjustment that is referred to as catabolic flexibility [46].

**Figure 6:**
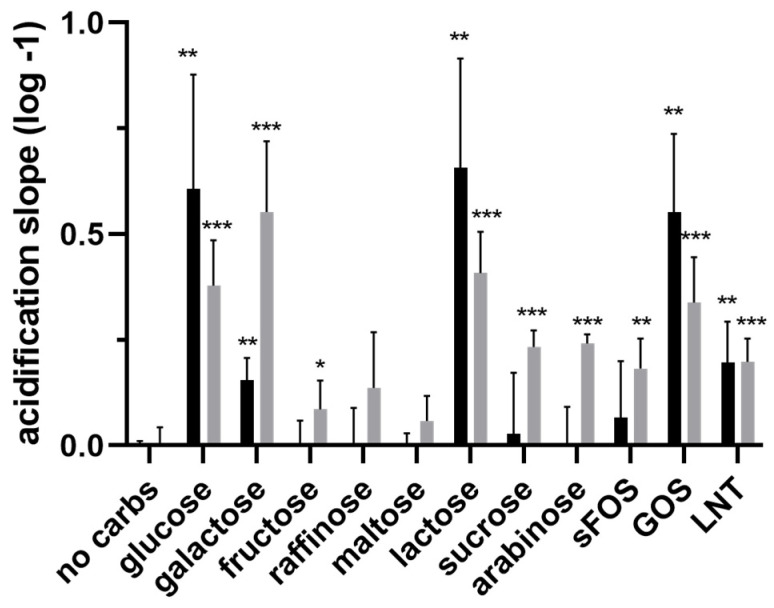
Initial acidification rates of glucose (black bars) or galactose (grey bars) mid-exponentially grown, translationally blocked *B. longum* NCC 2705 cells that were provided different carbohydrates. Values were extracted from the average logarithmic slopes obtained within the first 200 min of incubation after subtracting background acidification (no carbs) levels. Average acidification rates and standard deviation were calculated from biological triplicate measured in duplicate. Statistical analyses were performed using an unpaired multiple *t*-test comparing all carbohydrate conditions to the “no carbs” control (* *p* < 0.01, ** *p* < 0.001, *** *p* < 0.0001).

To confirm galactose growth mediated catabolic flexibility, glucose and galactose-grown cells were tested in an acidification assay using translationally blocked cells (using 5 μg/mL erythromycin) [42] and providing these with a range of carbohydrates that were previously shown to support NCC 2705 growth [20,38]. As expected, glucose grown cells were best adapted to import and metabolize glucose, whereas galactose-grown cells acidified at the highest rate when provided with galactose. Glucose grown cells could besides glucose also ferment galactose, lactose and GOS, and to a lesser extent also LNT. In contrast, galactose-grown cells could effectively ferment a substantially larger set of carbohydrates, including not only glucose, galactose, and lactose, but also fructose, sucrose, arabinose, scFOS, GOS and LNT, and albeit at a low rate, raffinose (Figure 6).

### 3.4. The Potential Implication or AraQ and MalR1 Lac-I Regulators

AraQ and MalR1 have previously been proposed as global bifidobacterial regulators [23]. We therefore determined the potential involvement of these two regulatory proteins in the orchestration of the distinct catabolic flexibility observed when comparing glucose or galactose-grown *B. longum* NCC 2705. Out of the 13 genomic loci predicted to be under AraQ transcription control [39], 9 were differentially expressed in our analysis. These loci encode genes that play prominent roles in the observed galactose-mediated adaptations, including the genes involved in the bifid-shunt (*BL0715* (*tal*), *BL0716* (*tkt*), *BL1363* (*gap*)) and those involved in the utilization of alternative carbon sources such as arabinose (*BL0272* (*araA*), *BL0274* (*araB*)), maltose (*BL0141* (*malE*)), galactose *(BL1359* (*galM*)) and 1,4-alpha-glucans (*BL0999* (*glgB*)). Conversely, the gene coding for a biotin-protein ligase (*BL1533*) was downregulated during growth on galactose, similar to the gene encoding AraQ itself (*BL0275*), which could reflect its capacity to both activate and repress transcription and its previously proposed autoregulation (Table 2).

A set of seven loci are predicted to be regulated by MalR1 in *B. longum* NCC 2705 [39]. Although the MalR1 encoding gene was not differentially expressed, four of its target loci were induced during growth on galactose, i.e., genes involved in maltose transport (*BL0141* (*malE*)), channeling of galactose and glucose into the bifid-shunt (*BL1630* (*pgm*), *BL1631* (*pgm*), *BL0279* (*pgi*)). Notably, two of the MalR1 target loci were induced when the strain was grown on glucose, i.e., genes involved in metabolization of amylose (*BL0527* (*malQ*)) and in transport of glucose (*BL1631* (*glcP*)) (Table 2). These findings tentatively support the involvement of AraQ and MalR1 in the control of the transcriptional adaptation elicited by the different carbon sources we studied and would thereby corroborate the role of these regulators in genome-wide gene expression regulation in *B. longum* NCC 2705.

### 3.5. Galactose-Grown B. longum NCC 2705 Cells Are More Acid-Stress Resistant

It has previously been suggested that smaller bifidobacterial cells display an enhanced stress tolerance [47,48]. In addition, changes in acetate:lactate ratio [49], and expanded fermentative repertoires (i.e., catabolic flexibility) [50,51] have been correlated to an increased pH resistance. Our NCC 2705 transcriptome data indicated that several stress related genes were upregulated when the strain was grown on galactose. For example, the extracytoplasmic function (ECF) sigma factor (σ^E^) (*BL1357*) and its putative anti-sigma factor (*BL1358*) were induced (Table S3), and have previously been demonstrated to be part of the heat stress response of NCC 2705 [52] and the bile stress response of *B. longum* BBMN68 [53]. Notably, also in several bacteria ECF-σ factors have been shown to be important regulators of diverse stress responses [54,55], including the response to acid stress in *Streptococcus mutans* [56]. In addition, a set of genes predicted to be involved in the synthesis of rhamnose rich exopolysaccharides (EPS) were transcriptionally increased in galactose-grown NCC 2705, including glucose-1-phosphate thymidylyltransferase (*BL0227*), dehydrorhamnose reductase (*BL0228*), EPS export genes (*BL0207*, *BL0208*), and extracellular glycosyltransferases (*BL0566*, *BL1674*) (Table S3). Homologues of these genes were previously shown to be upregulated upon acid stress in *B. longum* BBMN68 and were proposed to support the maintenance of cell-integrity under this condition [57]. Taken together, these changes may predict improved acid-stress resistance in galactose-grown NCC 2705 compared to their glucose grown counterparts. To assess this hypothesis, we performed a range of specific cell-stainings combined with flow-cytometry measurements.

**Figure 7:**
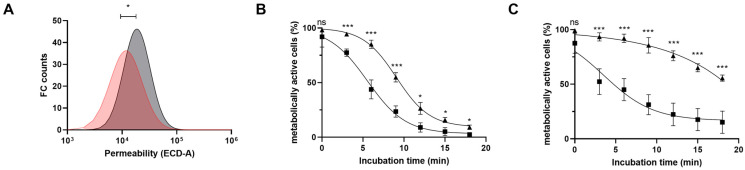
Cell permeability and acid sensitivity profile of *B. longum* NCC 2705 grown on glucose or galactose. (**A**) shows the propidium iodide (PI) stained cells distribution of mid-exponential phase harvested glucose (grey) or galactose (red) grown *B. longum* NCC 2705. Acid sensitivity profile of *B. longum* NCC 2705 grown on glucose (squares) or galactose (triangles) when exposed to pH 2.9 is depicted in (**B**,**C**). The loss of metabolically active cell populations (as reflected by the loss of esterase activity; changing from CFDA+ to CFDA−) collected from exponentially (**B**) or the stationary (**C**) phase of growth is represented by the average and standard deviation of biological triplicates. Logistic growth modelling was applied to each curve of CFDA− population (r^2^ > 0.90) and inversed data were plotted. Stars represent the adjusted *p*-value (*p*-value; ns > 0.05, * *p* < 0.05; *** *p* < 0.001) obtained upon multiple unpaired *t*-test for each time points.

Initially, using propidium iodide (PI)-stained cells, we observed that galactose-grown cells were significatively less permeable as compared to glucose grown cells (Figure 7A). Subsequently, resistance of these *B. longum* NCC 2705 cultures to low pH stress was assessed by following the fraction of metabolically active cells over time during low pH exposure. To establish an appropriate pH stress condition, glucose grown cells harvested during the stationary phase were exposed to a pH ranging from 2.9 to 3.5 (using phosphate-buffered solutions). This showed that the rate of metabolic activity loss in the population of cells was proportional to the pH they were exposed to. This is illustrated by the observation that more than 85% of the cells failed to show any metabolic activity after 18 min of incubation at pH 2.9, whereas only 44% had lost their metabolic activity upon exposure to pH of 3.5 for the same incubation time (Figure S2). Thus, glucose or galactose-grown *B. longum* NCC 2705 cells were harvested at the mid-logarithmic or stationary- phase of growth and exposed to a pH of 2.9 for a period of 18 min with regular assessment (every 3 min) of remaining metabolic activity in the population. A rapid loss of metabolic activity was observed in glucose grown cells, and this loss was faster in cells harvested from the mid-exponential (k_glu-expo_ = 0.43) as compared to stationary (k_glu-stat_ = 0.33) phase of growth (Figure 7B,C). In contrast, loss of metabolic activity of mid-exponentially harvested galactose-grown cells appeared to be delayed compared to their glucose grown counterpart, i.e., the decrease of metabolic activity only started after 6 min of pH 2.9 exposure. Moreover, galactose-grown cells harvested during the stationary phase appeared more stress resistant because the loss of metabolic activity was even further delayed and a substantial proportion of the cells maintained their metabolic activity (~60%) during the entire assay period. Similar to what was observed for glucose grown cells, metabolic activity loss in galactose-grown cells declined at a faster rate in cells obtained from the exponential—(k_gal-expo_ = 0.49) compared to the stationary—(k_glu-stat_ = 0.33) (Figure 7B,C). These results showed that, as expected, stationary-phase harvested cells were more resistant compared to logarithmic-phase harvested cells irrespective of the carbon source they were grown on. Moreover, these results show that irrespective of the growth phase of harvesting, glucose-grown cells not only display a higher intrinsic permeability (Figure S3) but also are more susceptible to pH stress (pH of 2.9) as compared to their galactose-grown counterparts.

## 4. Conclusions & Discussion

Our results highlight important physiological differences between cells of *B. longum* NCC 2705 grown on glucose or galactose as the sole carbon source. Compared to glucose, galactose-grown NCC 2705 displayed a reduced growth rate and a smaller cell size. Gene transcription analysis revealed metabolic adaptations in glucose growing cells that may reflect the higher growth rate on this sugar, such as the upregulation of nucleotide biosynthesis and ATP generation. On the other hand, a reduced growth rate on galactose was accompanied by an expanded catabolic flexibility phenotype, where a variety of transcriptional changes related to carbon source import and central energy metabolism supported our experimental findings. The two growth conditions were associated with a modulation of the metabolic end-products, including an increased acetate:lactate ratio and the production of ethanol and formate by cells grown on galactose. Moreover, we demonstrated that galactose-grown *B. longum* NCC 2705 were less permeable and displayed an enhanced acid-stress resistance as compared to glucose grown cells.

Catabolic flexibility, a bacterial physiological status under which a wider range of substrates can be utilized [46], is often induced in cells with a reduced growth rate [45]. Furthermore, growth rate reduction has been shown in different bacterial species to govern their resistance to different stresses [26,27,28,58,59]. A few studies using bifidobacteria have evidenced some of these features. For example, *B. longum* BBMN68 pre-exposed to mild acid stress exhibited an enhanced level of stress resistance, as well as increased expression of genes involved in carbohydrate metabolism and energy production [50]. Another study showed that acid-resistant derivatives of *B. longum* and *B. catenulatum* displayed increased catabolic flexibility [51]. Bile salt resistant derivatives of *B. bifidum* CECT 4549 and *B. infantis* CECT 4551 displayed an enhanced fructose-6-phoshoketolase (F6PPK) activity that was accompanied by an increased ratio of the metabolic end-products acetate:lactate [60]. Finally, acid resistance in *B. breve* BB8 was accompanied by reduced expression of genes involved in the production of lactic acid and increased expression of genes associated with ethanol production [49]. In this study, we conducted a head-to-head comparison of *B. longum* NCC 2705 grown on glucose or galactose. This allowed us to establish that galactose-grown cells displayed a reduced growth rate accompanied by expansion of catabolic flexibility, modified central carbon metabolism (increased ethanol and acetate:lactate ratio) and increased pH stress resistance. The transcriptome analysis we performed on these cells supports most of the observed phenotypic changes and points to the existence of a catabolite repression-like regulatory mechanism. We propose this mechanism to involve the two global gene expression regulators known in *B. longum* NCC 2705 (AraQ and MalR1 [23,24]) that control genes associated with the enhanced catabolic flexibility as well as the modified central carbon metabolism we observed in galactose-grown cells. The transcriptome data imply that AraQ itself is produced at a lower level during growth on galactose as compared to glucose, suggesting that this decrease in AraQ expression may relieve the repression of several target genes, thus supporting its global role in carbon-source regulation in *B. longum* NCC 2705. Lanigan and colleagues [23] suggested that a carbohydrate or a metabolic intermediate may act as an effector molecule to enhance the binding capacity of these transcription factors. They did not succeed in identifying the exact nature of this activator, but we cannot exclude that galactose, or one of its metabolic intermediates may play such a role. In addition, the transcriptome revealed that galactose induced expression of the ECF σ^E^ (*BL1357*) and its postulated anti-sigma factor (*BL1358*) encoding genes, which we suggest play a role in the elevated pH stress tolerance we observe. This hypothesis is in agreement with observations in other bacteria [61], such as *E. coli* [26], *B. subtilis* [62] and *Streptococcus mutans* [56], where homologues of this alternative sigma factor were shown to control a large set of general stress response proteins and contribute to tolerance to a variety of stress conditions, including pH stress [56]. Therefore, we postulate that the induction of ECF σ^E^ in galactose-grown NCC 2705 provides protection to a wider range of stress conditions, potentially including heat [52] and oxidative stress [63].

Galactose-grown NCC 2705 produced elevated levels of ethanol, which contributes to NAD+ regeneration [15,64]. In other species, such as *L. lactis*, the reduction of growth rate has been associated with a change in fermentation end products, including ethanol formation, and was additionally accompanied by an increased secondary metabolism activity [58]. Intriguingly, molecules that have been demonstrated to be important for the survival or host-interaction capacity of bifidobacteria, such as serpin [20] and sortase-dependant pili [65,66,67] have been shown to be regulated by different carbohydrates, warranting further research into the relationship between reduced growth rates and the production of bifidobacterial niche and effector molecules.

Overall, our results provide evidence for a prominent role of carbohydrate substrates in eliciting a broad spectrum of physiological changes in *B. longum* NCC 2705. The changes induced by growth on galactose are potentially very important for the adaptation of NCC 2705 to the gut environment, particularly in the early infancy, where bifidobacteria are exposed to and catabolize different HMOs, many of which contain galactose moieties [8]. Moreover, glucose is likely to be much less abundant in the infant colon. The main source of glucose in human milk is in the form of lactose that is hydrolyzed by intestinal lactase, thereby releasing glucose which is rapidly absorbed in the small intestine of the infant [68]. Therefore, the galactose growth associated phenotype of NCC 2705 may arguably mimic the strain physiological status of the infant gut, where catabolic flexibility and stress resistance enhancement are likely to increase the strain fitness. More generally, reduced growth rates (e.g., as induced by growth on galactose) may lead to the production of higher levels of inducible molecules in the gut (i.e., niche factors and effector molecules).

Beyond their relevance to the bifidobacterial lifestyle in the gut, our results might have important implications for probiotic production and formulation. Although glucose is an attractive carbon source for industrial manufacturing due to its capacity to support fast growth, it may lead to the production of a bacterial biomass not optimally adapted to the intestinal milieu. Biomass produced using alternative carbohydrates as substrate (e.g., galactose) may display more appropriate attributes, preparing the cells for the challenging gastrointestinal conditions. As an alternative, the formulation of *Bifidobacterium* strains in combination with selected prebiotics (e.g., galacto-oligosaccharides or HMOs) that are rich in galactose moieties may increase the probiotic in situ adaptation. These considerations indicate that carbohydrate substrate selection for industrial production, or the prebiotic added in the final synbiotic formulation, may modulate the physiology of the probiotic bacteria, which may in turn affect their in situ survival and efficacy.

## Figures and Tables

**Table 1:** Growth characteristics of *B. longum* NCC 2705 grown in DasGip bioreactors with 1% of glucose or galactose. Maximal acidification slope was obtained by modeling inversed pH values. Maximal growth rate was retrieved from the modelling of the OD curves. An average CFU:OD ratio was calculated based on all data obtained before the decay in CFU. The estimated doubling time was obtained by modeling the increase in theoretical CFU through time. Average cell length represents the average of all data (early-exponential, mid-exponential and stationary harvested cells) obtained for the different conditions.

	GLUCOSE		GALACTOSE	
	OD-Based	CFU-Based	OD-Based	CFU-Based
**MAX CELL DENSITY ± SEM (OD600; CFU/ML)**	5.87 ± 0.12	2.80 × 10^8^ ± 1.86 × 10^7^	3.80 ± 0.06	7.80 × 10^8^ ± 1.22 × 10^8^
**AVERAGE MAX GROWTH RATE μ ± SEM (1/H)**	0.67 ± 0.05	-	0.35 ± 0.05	-
**ESTIMATED AVERAGE DOUBLING TIME (1/H)**	-	1.05	-	2.06
**AVERAGE ± SEM CFU:OD**	5.93 × 10^7^ ± 1.81 × 10^7^		2.54 × 10^8^ ± 4.28 × 10^7^	
**AVERAGE CELL LENGTH ± SEM (μM)**	2.47 ± 0.06		1.57 ± 0.03	
**MAX ACIDIFICATION SLOPE (1/H)**	−0.57 ± 0.07		−0.39 ± 0.03	
**AVERAGE FINAL PH ± SEM**	4.51 ± 0.04		4.50 ± 0.05	

**Table 2:** Differential expression values of genes that are predicted to belong to the AraQ (BL0275) and MalR1 (BL0142) regulons in *B. longum* NCC 2705, as presented in RegPrecise. Genes showing a significant differential expression are indicated in bold. Induction during galactose growth is depicted by positive log2FC, whereas negative log2FC values represent induction during glucose growth.

	Locus Tag	Gene Name	LOG2FC	FDR Q-Value	Function
**ARAQ Regulated Loci**	**BL0141**	***malE***	**0.728**	**0.007**	**Maltose/maltodextrin ABC transp., substrate binding protein**
	**BL0272**	***araA***	**1.218**	**0.006**	**L-arabinose isomerase**
	BL0273	*araD*	ND	NA	L-ribulose-5-phosphate 4-epimerase
	**BL0274**	***araB***	**1.932**	**0.003**	**Ribulokinase**
	**BL0275**	***araQ***	**−1.106**	**0.037**	**Transc. regulator of central carb. metabolism, LacI family**
	**BL0715**	***tal***	**0.705**	**0.006**	**Transaldolase**
	**BL0716**	***tkt***	**0.526**	**0.011**	**Transketolase**
	BL0988	*pyk*	0.387	0.057	Pyruvate kinase
	**BL0999**	***glgB***	**0.697**	**0.002**	**GH13 glycosyl hydrolase**
	BL1000	*-*	1.388	0.091	Response regulator of two-component system
	BL1001	*-*	0.368	0.391	histidine kinase sensor of two-component system
	BL1022	*eno*	0.031	0.928	**Enolase**
	BL1308	*ldh*	−0.085	0.785	L-lactate dehydrogenase
	**BL1359**	***galM***	**2.006**	**0.002**	**Aldose 1-epimerase**
	**BL1363**	***gap***	**0.912**	**0.000**	**NAD-dependent glyceraldehyde-3-phosphate dehydrogenase**
	**BL1531**	***-***	**1.185**	**0.000**	**Regulator of polyketide synthase expression**
	**BL1532**	***-***	**1.581**	**0.000**	**hypothetical protein**
	**BL1533**	***-***	**−3.372**	**0.000**	**Biotin-protein ligase**
	BL1570	*malQ1*	0.168	0.590	4-alpha-glucanotransferase
**MALR1 REGULATED LOCI**	**BL0279**	***pgi***	**2.079**	**0.000**	**Glucose-6-phosphate isomerase**
	BL0597	*glgP*	−0.065	0.841	Glycogen phosphorylase
	BL0142	*malR1*	0.370	0.252	Transcr. regulator of maltose/maltodextrin utilization, LacI family
	**BL0141**	***malE***	**0.728**	**0.007**	**Maltose/maltodextrin ABC transp., substrate binding protein**
	**BL1630**	***pgm***	**1.006**	**0.001**	**Phosphoglucomutase (EC 5.4.2.2)**
	**BL1631**	***glcP***	**−0.930**	**0.001**	**D-Glucose-proton symporter**
	**BL0527**	***malQ***	**−1.834**	**0.000**	**4-alpha-glucanotransferase (amylomaltase)**
	BL0528	*malR2*	−0.349	0.664	Transcr. regulator of maltose/maltodextrin utilization, LacI family
	BL0529	*aglA*	−0.925	0.063	Alpha-glucosidase
	BL0143	*malF*	0.713	0.222	Maltose/maltodextrin ABC transporter
	BL0144	*malG*	0.699	0.263	Maltose/maltodextrin ABC transporter

## Data Availability

RNAseq data used in this study are deposited at NCBI (Sequence Read Archive) in the Bioproject PRJNA936080.

## Supplementary Materials

The following supporting information can be downloaded at: https://www.mdpi.com/article/10.3390/microorganisms11030588/s1, Figure S1: Gene Ontology (GO) functions enriched in glucose or galactose upregulated genes; Figure S2: Metabolic activity of *B. longum* NCC 2705 stationary grown glucose cells exposed to pH 2.9, pH 3.0, pH 3.2 and pH 3.5 for a duration of 18 min at 37 °C; Figure S3: *B. longum* NCC 2705 population distribution upon exposure to a pH of 2.9. for 12 min; Table S1: List of *B. longum* NCC 2705 genes enriched in the glucose grown cells and significatively associated to different Gene Ontologies (GO) upon GO enrichment analysis; Table S2: List of *B. longum* NCC 2705 genes enriched in the galactose-grown cells and significatively associated to different Gene Ontologies (GO) upon GO enrichment analysis; Table S3: List of differentially expressed *B. longum* NCC 2705 genes upon growth on galactose or glucose as sole carbon source.
