# Supplementary figures and images for: The Pleiotropic Effects of Carbohydrate-Mediated Growth Rate Modifications in Bifidobacterium longum NCC 2705
Source: Microorganisms. 2023 Feb 26;11(3):588. doi: 10.3390/microorganisms11030588 (PMC10059941; doi:10.3390/microorganisms11030588)

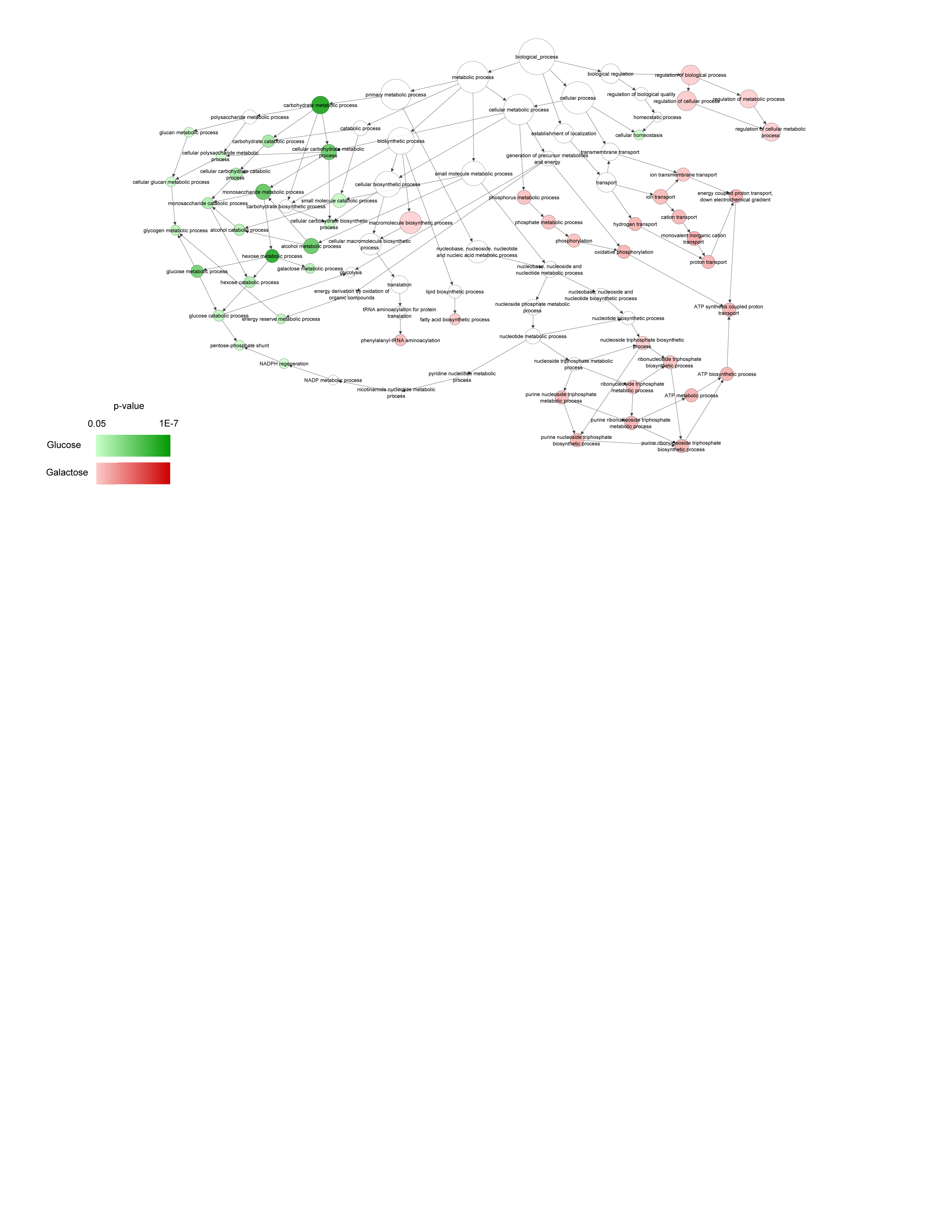

Supplement: Supplementary file 1 [file microorganisms-11-00588-s001.zip › FigureS1.png]

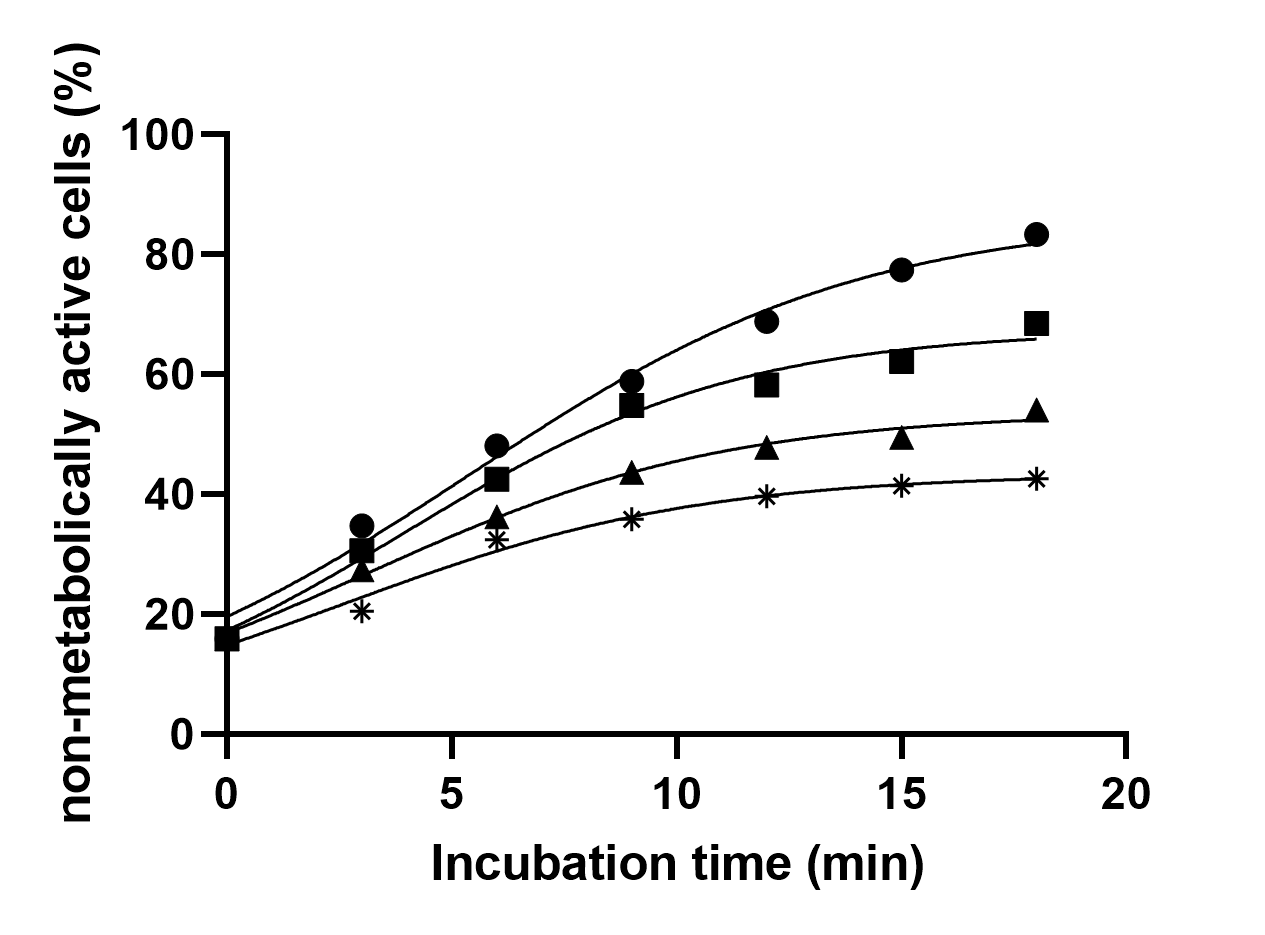

Supplement: Supplementary file 1 [file microorganisms-11-00588-s001.zip › FigureS2.png]

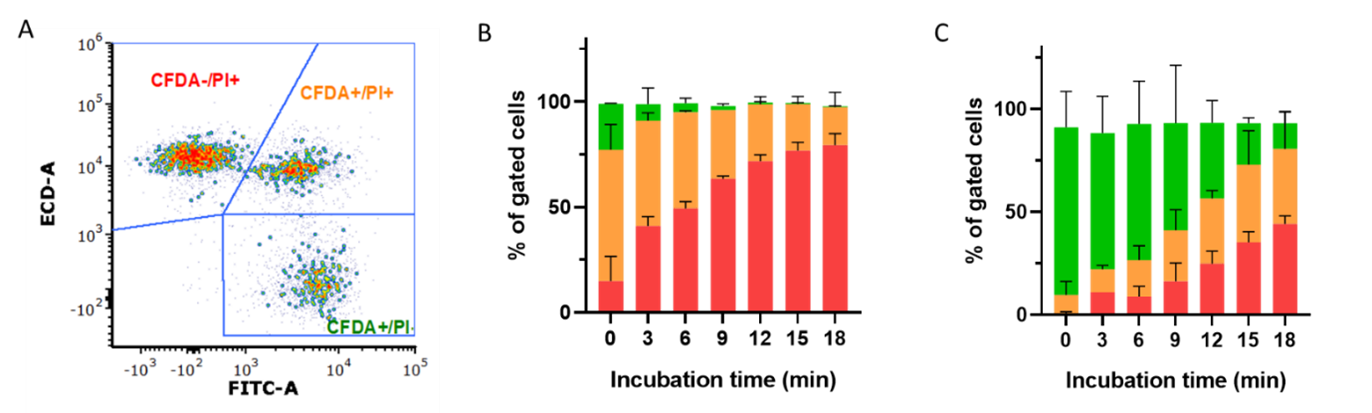

Supplement: Supplementary file 1 [file microorganisms-11-00588-s001.zip › FigureS3.png]
